# Transcriptome Profiling Identifies Differentially Expressed Genes in Postnatal Developing Pituitary Gland of Miniature Pig

**DOI:** 10.1093/dnares/dst051

**Published:** 2013-11-26

**Authors:** Lei Shan, Qi Wu, Yuli Li, Haitao Shang, Kenan Guo, Jiayan Wu, Hong Wei, Jianguo Zhao, Jun Yu, Meng-Hua Li

**Affiliations:** 1 Key Laboratory of Animal Ecology and Conservation Biology, Institute of Zoology, Chinese Academy of Sciences, Beijing 100101, China; 2 CAS Key Laboratory of Genome Sciences and Information, Beijing Institute of Genomics, Chinese Academy of Sciences, Beijing 100101, China; 3 College of Life Sciences, University of Chinese Academy of Sciences, Beijing 100049, China; 4 Department of Laboratory Animal Science, College of Basic Medical Sciences, Third Military Medical University, Chongqing 400038, China; 5 The State Key Laboratory of Reproductive Biology, Institute of Zoology, Chinese Academy of Sciences, Beijing 100101, China

**Keywords:** miniature pig, pituitary gland, whole transcriptome analysis, RNA-sequencing

## Abstract

In recent years, Tibetan pig and Bama pig are popularly used as animal models for medical researches. However, little genomic information is available for the two breeds, particularly regarding gene expression pattern at the whole-transcriptome level. In this study, we characterized the pituitary transcriptome profile along their postnatal developmental stages within and between the two breeds in order to illustrate the differential dynamics and functions of differentially expressed genes. We obtained a total of ∼300 million 80-bp paired-end reads, detected 15 715 previously annotated genes. Most of the genes (90.33%) were shared between the two breeds with the main functions in metabolic process. Four hormone genes (*GH*, *PRL*, *LHB*, and *FSHB*) were detected in all samples with extremely high levels of expression. Functional differences between the three developmental stages (infancy, puberty and adulthood) in each breed were dominantly presented by the gene expressions at the first stage. That is, Bama pig was over-represented in the genes involved in the cellular process, while Tibetan pig was over-represented in the genes represented by the reproductive process. The identified SNPs indicated that the divergence between the miniature pig breeds and the large pig (Duroc) were greater than that between the two miniature pig breeds. This study substantially expands our knowledge concerning the genes transcribed in the pig pituitary gland and provides an overview of pituitary transcriptome dynamics throughout the period of postnatal development.

## 1. Introduction

The swine (*Sus scrofa domesticus*), particularly the miniature breeds, has been used as an important biomedical model organism for human disease studies because of the similarity in size, anatomy and physiology, as well as in organ development and disease progression with those of human.^1^ Bama pig and Tibetan pig, two Chinese indigenous miniature pig breeds inhabiting in Guangxi province and Tibetan plateau, respectively, are increasingly developed as animal models for medical studies in recent years. The relevant applications included the utilization of them as a model for human drug evaluation, xenotransplantation and diverse disease studies, and the developments of induced pluripotent stem (iPS) cell lines.^2–9^ Despite of their significant role in medical studies, our knowledge of whole-genome information at the transcript level (i.e. transcriptome) in these two breeds remains limited. For instance, patterns of transcriptome divergence between different postnatal developmental stages within and between the two breeds, which are from regions of different altitudes (Bama pig, 600–800 m above sea levels (m.a.s.l.); Tibetan pig, 2500–4300 m.a.s.l.), are poorly studied.

With the rapid development in genomics, it is essential for a model organism to create deep genomic sequence data^10–12^ as well as detailed and integrated transcriptome data^13,14^ for tissues across developmental stages. Although the pig genome and transcriptome profiles have been characterized in some breeds in recent years,^11,15–20^ the transcriptome profiling still need further improvement in the characterization of both more tissues (organs) and more different developmental stages of these organs. For example, the transcriptome profiling of swine pituitary gland at different developmental stages would be an important add-in to a comprehensive profile of the porcine transcriptome.

The pituitary gland, as a ‘master’ endocrine organ, receives signals from the brain and uses these messages to produce hormones that regulate developmental and physiological processes including growth, pubertal development, metabolic status, reproduction, lactation, and the capacity to cope with stress.^21,22^ Hence, profiling the transcriptome patterns of pituitary gland along the developmental process may enable us to understand not only the physiological functions of genes in the organ but also how the expression dynamics shapes the development characteristics at the individual or breed level. In this study, we investigated the anterior pituitary transcriptome in Bama (BM) pig and Tibetan (TN) pig at three representative stages: infancy (1.5-month), puberty (4-month), and adulthood (6-month for BM, and 8-month for TN) using Solexa RNA-seq technologies. These results allowed us to establish a general overview on the differential dynamics of gene expression in the swine pituitary gland, and further investigate the underlying functional differences in differentially expressed genes among the postnatal development stages and between the breeds.

## 2. Materials and methods

### 2.1. The animal and sampling

Three Bama male pigs and three Tibetan male pigs were included in this study (Supplementary Data). The Bama pigs were housed in a miniature pig farm in Chongqing city, southwest China. The farm was managed by the Department of Laboratory Animal Science of College of Basic Medical Sciences in the Third Military Medical University. They are the descendants of sows and boars obtained from the original stocks at Bama County, Guangxi Province. The Tibetan pigs were housed in a pig farm in Nyingchi Prefecture, east of Tibet Autonomous Region, China, and the farm was managed by the College of Agriculture and Animal Husbandry of the Tibet University. They are from the pure stocks captured in the wild in originally distributional areas in Tibet. The three Bama male pigs consist of one 1.5-month-old (BM1, infancy, weight = 5.8 kg, 7 January 2010), one 4-month-old (BM4, puberty, weight = 11 kg, 22 January 2010), and one 6-month-old (BM6, adulthood, weight = 20 kg, 22 January 2010). The three Tibetan male pigs include one 1.5-month-old (TN1, infancy, weight = 8.75 kg, 29 January 2010), one 4-month-old (TN4, puberty, weight = 12.5 kg, 29 January 2010), and one 8-month-old (TN8, adulthood, weight = 52.5 kg, 29 January 2010). The status of sexual maturation in pubertal and adult pigs was examined by the pig farmers by their long-term experiences. Pubertal pigs are defined as the pigs that are capable of sexual reproduction, i.e. the first ejaculation. Adult pig refers to the pig reaching to body mature. Procedures involving animals were approved by the Committee for Animal Experiments of the Institute of Zoology, Chinese Academy of Sciences, China (No. IOZ13015) and complied with the Laboratory Animal Management Principles of China. The animals were euthanized by intravenous injection of pentobarbital sodium (150 mg/kg). The tissues were sampled immediately after death. The tissues were washed with saline, then cut into small pieces and saturated in Sample Protector (TaKaRa Code: D311A, TaKaRa Biotechnology (Dalian) Co., Ltd., China) and stored in −80°C refrigerator for preservation before use. TrIzol (Life Technologies (Beijing), China) was used to isolate total RNA following the manufacturer's protocols.

### 2.2. cDNA library construction and sequencing

Sequencing libraries were prepared according to the manufacturer's instructions (Illumina, USA). Poly(A) mRNA was isolated from the total RNA through two purification rounds using poly-T oligo-attached magnetic beads, and was further fragmented. The first-strand cDNA was generated using reverse transcriptase and random hexamer primers. Following the second-strand cDNA synthesis and adaptor ligation, 300–400-bp cDNA fragments were isolated. The paired-end cDNA libraries were then prepared in accordance with Illumina's protocols and sequenced on Illumina GAII platform to generate 80-bp paired-end reads. The Illumina data processing pipeline version 1.4 was applied in image analysis and base calling.

### 2.3. Bioinformatics analysis

#### Reads trimming and alignment

After trimming low-quality reads using FASTX Toolkit v0.0.13, the high-quality paired reads were aligned against the reference genome of *Sus Scrofa* (Sscrofa9.2; http://www.ncbi.nlm.nih.gov/assembly/111518/) using the Burrows–Wheeler Alignment Tool^23^ (BWA) with allowing up to four mismatches and other default options. Alignments were also performed against the junction database (for 75 bp × 2) constructed by permutation and combination of exons for each gene. We counted the number of reads mapped to exons, introns, splice junctions, intron–exon adjacent regions, and intergenic regions of the annotated genes, respectively.

#### Gene annotation and expression analysis

The gene expression levels were measured as numbers of reads per kilobase of exon model in a gene per million mapped reads (RPKM).^14^ Genes (RPKM > 0) that were detected commonly in all the six samples were assigned as co-genes. To annotate the function of these co-genes, we conducted Gene Ontology (GO) analysis^24^ at the third level for each of the three main categories: ‘biological process’, ‘cellular component’, and ‘molecular function’ using in-house Perl scripts, which, by our experiences in data analysis, can explicitly and properly illustrate the functions of these genes and cluster the genes into categories based on their functions.

Differentially Expressed Genes (DEGs) between different stages in each breed were identified with a *R* package named ‘DEGseq’.^25^ We took those with the combination of ‘at least two mapped reads, FDR (false discovery rate) ≤0.001 and the log_2_-fold changes ≥1’ of a gene at one stage in comparisons to the other two stages as stage-specific highly upregulated genes. We averaged the RPKM values at the three stages of each gene, and calculate the log_2_-fold changes of each gene between the two breeds. Genes with log_2_-fold change ≥1 or ≤ −1 were assigned as the BM-specific (BHU) or TN-specific highly upregulated (THU) genes. These stage- or breed-specific highly upregulated genes were annotated in GO terms involved in biological process (*P*-value < 0.05). To reveal the expression profile of pituitary hormone genes among the developmental stages, we calculated the fold-change ratios in gene expression levels based on RPKM between the two adjacent stages in each breed.

#### SNP identification

RNA-seq technology has the advantages of quickly and reliably discovering single-nucleotide polymorphisms (SNPs). We conducted transcriptome comparisons between the two pig breeds and between each and the reference genome sequence of Duroc. SNPs were identified from the consensus sequence among the three transcriptome datasets within each of the breeds, and were referred that sequence within each breed to the reference genome sequence, respectively, using Sequence Alignment/Map tools version 0.1.17^26^ (SAMtools; http://samtools.sourceforge.net) with the following criteria: nucleotide with reads mapping quality score ≥60; consensus quality score ≥30; sequence depth ≥10. Further annotation of the SNPs was made with in-house Perl scripts.

## 3. Results

### 3.1. High-throughput sequencing and reads mapping

**Figure 1.**
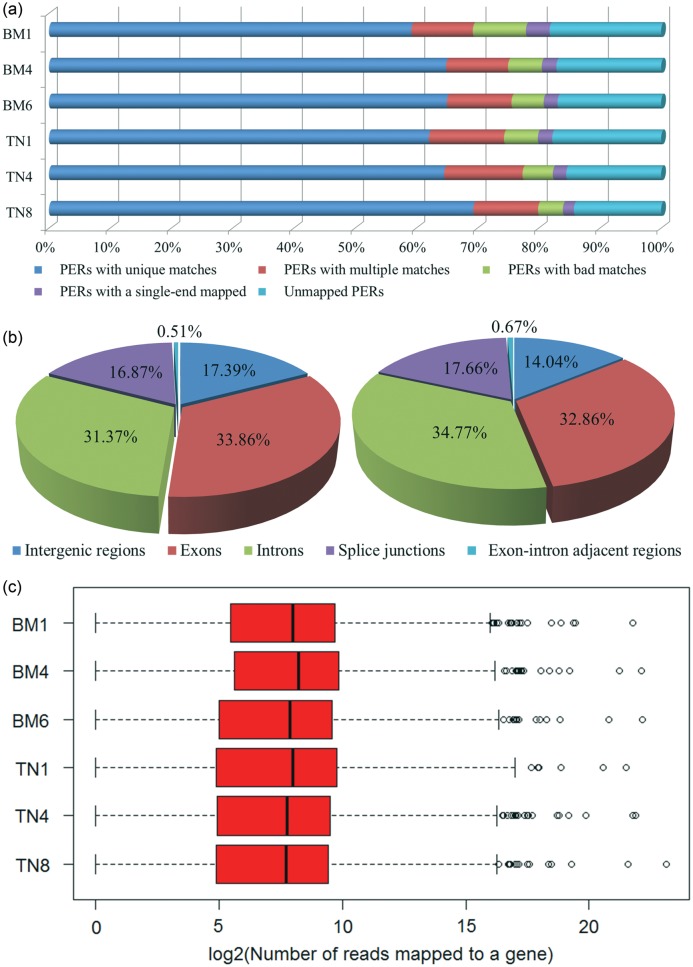
Summary of RNA-seq mapping data. (a) overall mapping results of paired-end reads (PERs); (b) distribution of reads in structural features of genes annotated in pig genome, Bama (Left pie) and Tibetan (Right pie) pigs; (c) box-and-whisker plots show log_2_ transformed values of the number of unique reads per gene at the six RAN-seq data. The black line in the box represents the median.

We obtained a number of ca 53.21, 49.05, 42.07, 47.05, 57.50, and 51.68 millions of raw reads for BM1, BM4, BM6, TN1, TN4, TN8, respectively (Table 1). The total length of the reads was over 24 gigabases (Gbs), representing around 8.9-fold of the whole pig genome (ca 2.7 Gbs). After discarding low-quality reads, 73.84–89.66% of the raw reads were remained as high-quality reads. Of which, 59.18–69.3% was mapped uniquely to the pig reference genome, 10.05–12.81% showed multiple matches (hereafter the ‘good matches’ referred to the unique and multiple matches), 14.25–18.23% had no matches, and the remaining 7.14–12.54% were further filtered as ‘bad’ matches at a coarser level or matched with only one end of the paired-end reads (Fig. 1a). The genomic distribution patterns of uniquely mapped reads showed that a majority of the reads located in annotated genic regions, with an average of 82.61% for BM and 85.96% for TN (Fig. 1b). 30.21–37.72% completely fell in annotated exons, 27.47–42.78% fully located in introns, 14.01–19.59% positioned to splice junctions, 0.46–1.04% overlapped with exons and introns. The remaining 8.25–23.16% of the uniquely mapped reads was assigned to intergenic regions. Furthermore, the number of uniquely mapped reads assigned to a known gene (at least with one mapped read) varied substantially in the samples, ranging from one to over three million with a median of 200–300 reads for each sample (Fig. 1c).

**Table 1.** Summary of reads and matches

Reads/matches	BM1	BM4	BM6	TN1	TN4	TN8	Total
Total raw reads	53 205 644	49 051 250	42 069 878	47 045 734	57 500 596	51 681 368	30 0554 470
High-quality paired-end reads (PERs)	44 339 466	42 852 634	37 719 878	37 445 200	42 458 576	41 212 194	246 027 948
Unmatched PERs	8 082 220	7 332 163	6 382 924	6 665 295	6 585 149	5 874 558	40 922 309
PERs with a single-end matches	1 713 028	1 028 699	858 300	878 857	928 819	743 991	6 151 694
PERs with ‘bad’ matches	3 846 656	2 362 260	1 980 354	2 070 918	2 101 984	1 670 785	14 032 957
PERs with ‘good’ matches	30 697 562	32 129 512	28 498 300	27 830 130	32 842 624	32 922 860	184 920 988
PERs with multiple matches	4 456 260	4 334 066	3 982 408	4 595 348	5 439 012	4 362 342	27 169 436
PERs with unique matches	26 241 302	27 795 446	24 515 892	23 234 782	27 403 612	28 560 518	157 751 552
PERs in intergenic regions	6 078 183	4 216 290	3 392 568	4 437 032	4 044 245	2 357 067	24 525 385
PERs completely in exons	8 834 290	9 509 548	8 261 841	7 119 986	10 336 824	8 628 482	52 690 971
PERs fully in introns	7 521 316	8 800 482	8 285 426	7 916 921	7 528 423	1 221 6802	52 269 370
PERs aligned to splice junctions	3 675 337	5 135 401	4 440 746	3 642 109	5 367 822	5 059 847	27 321 262
PERs aligned to exon–intron junctions	132 176	133 725	135 311	118 734	126 298	298 320	944 564

### 3.2. Global gene expression analysis

**Figure 2.**
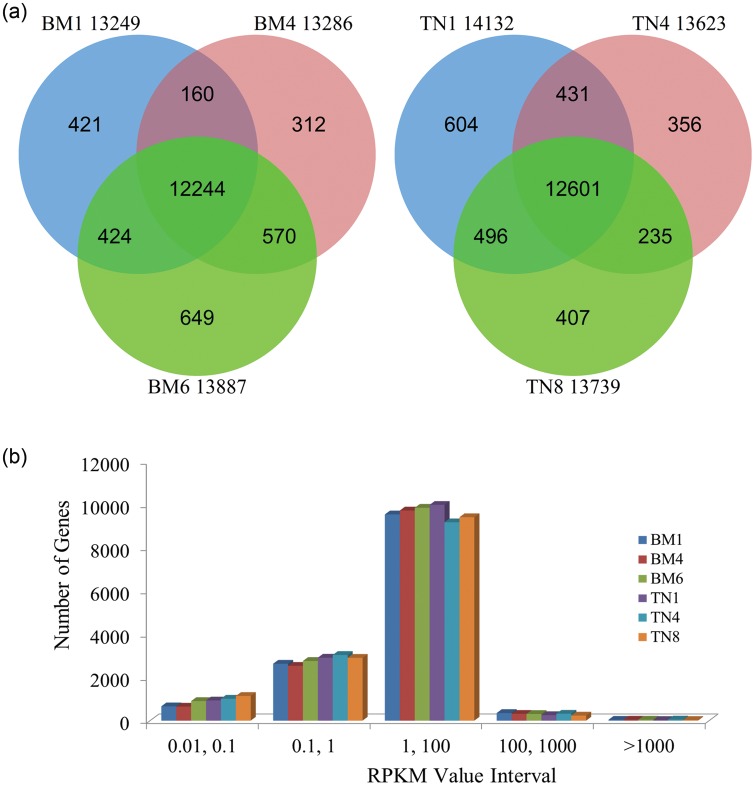
(a) Annotated genes detected among BM1, BM4, and BM6 (Left diagram) and among TN1, TN4, and TN8 (Right diagram); (b) the number of detected genes with different expression levels against the range of RPKM values.

A total of 15 715 annotated genes were detected with RPKM > 0 in the two breeds at all the three stages (Supplementary Table S1). Of these genes, a large proportion (90.33%, 14 195/15 715) was detected commonly to the two breeds, whereas a much smaller proportion (3.72%, 585/15 715; 5.95%, 935/15 715) were represented exclusively in Bama and Tibetan pig (Supplementary Table S2). With respect to the different developing stages, 13 249, 13 286, 13 887, 14 132, 13 623, and 13 739 genes were detected from each of the transcriptome datasets for BM1, BM4, BM6, TN1, TN4, and TN8, respectively (Fig. 2a). In Bama pig, a total of 12 244 genes were represented at all three stages. 160, 570, and 424 genes were identified commonly between each pair of stages, while 421, 312, and 649 genes were discovered exclusively for BM1, BM4, and BM6, respectively. Similarly, 12 601 genes were represented at all three stages in Tibetan pig. 431, 235, and 496 genes were expressed commonly between each pair of stages, while 604, 356, and 407 genes were discovered uniquely in TN1, TN4, and TN8, respectively. It was worth noting that the numbers of genes detected in the samples were very similar between the two breeds in terms of RPKM (Fig. 2b).

### 3.3. Expression characterization in pituitary transcriptome

In total, 11 844 co-genes were detected and were undergoing GO analysis, and 9381 of which were assigned to 431 GO terms. The number of genes allocated to different terms ranged from 1 to 6507. Among the significantly enriched GO terms (*P*-value < 0.05), the top 10 from each GO category with the highest numbers of co-genes were shown in Table 2, mainly being involved in metabolic process, positioning to cell part, and functioning in protein binding.

**Table 2.** The top 10 terms with highest number of co-genes in each of the three main categories of gene ontology (GO) at a significant level of 0.001 (*P* < 0.001)

No.	Terms at the third level of GO	GO Accession	No. of genes
	*Biological process*		
1	Primary metabolic process	GO:0044238	3738
2	Cellular metabolic process	GO:0044237	3608
3	Macromolecule metabolic process	GO:0043170	3167
4	Biosynthetic process	GO:0009058	2005
5	Nitrogen compound metabolic process	GO:0006807	1922
6	Regulation of metabolic process	GO:0019222	1457
7	Establishment of localization	GO:0051234	1408
8	Transport	GO:0006810	1392
9	Multicellular organismal development	GO:0007275	1170
10	Anatomical structure development	GO:0048856	1090
	*Cellular component*		
1	Cell part	GO:0044464	6507
2	Intracellular	GO:0005622	5134
3	Intracellular part	GO:0044424	4497
4	Intracellular organelle	GO:0043229	3811
5	Membrane-bounded organelle	GO:0043227	3372
6	Organelle part	GO:0044422	1561
7	Intracellular organelle part	GO:0044446	1547
8	Protein complex	GO:0043234	1106
9	Non-membrane-bounded organelle	GO:0043228	978
10	Organelle lumen	GO:0043233	523
	*Molecular binding*		
1	Protein binding	GO:0005515	4417
2	Ion binding	GO:0043167	1789
3	Nucleic acid binding	GO:0003676	1660
4	Hydrolase activity	GO:0016787	1365
5	Nucleotide binding	GO:0000166	1331
6	Transferase activity	GO:0016740	1054
7	Nucleoside binding	GO:0001882	1025
8	Binding	GO:0005488	523
9	Catalytic activity	GO:0003824	498
10	Ligase activity	GO:0016874	236

To further characterize the unique gene expression profile in the pituitary, we included 61 genes that recurrently appeared in the top 100 most highly transcribed genes of each sample, and then grouped them into five categories in terms of their functions (Supplementary Table S3). The first category included the most actively transcribed genes for pituitary hormones (growth hormone (*GH*), glycoprotein hormones alpha (*CGA*), prolactin *(PRL*), Lutropin subunit beta (*LHB*), and follicle stimulating hormone beta (*FSHB*), which were expressed specifically and specify the pituitary gland. The second and third category contains ribosomal protein genes and mitochondrial redox carrier protein genes, respectively, which were intensively expressed due to the protein synthesis and energy requirement for the animal development. The fourth category contains genes encoding proteins with divergent functions, such as Neuronatin (*NNAT*) was reported to participate in the maintenance of segment identity in pituitary development,^27^ and considered to be associated with the pituitary adenomas.^28^ Chromogranin B (*CHGB*) is a neuroendocrine secretory granule protein playing an important role during vesicle biogenesis^29^ and secretion of hormones to exocytosis via the regulatory pathway.^30^ Most of the other genes were not expressed specifically in the pituitary, and thus were considered housekeeping genes, and some of these genes may employ as internal standards, such as *ACTB* and *GAPDH*.^31^ The fifth category contains genes annotated to be miRNA in *Ensembl*.

### 3.4. Expressional differences among the developing stages

We identified 1123, 64, 172, 1379, 210, and 132 DEGs that were stage-specific highly upregulated genes for BM1, BM4, BM6, TN1, TN4, and TN8, respectively (Supplementary Fig. S1). In GO enrichment analysis, a total of 80 GO terms involved in biological process were significantly (*P* < 0.05) enriched across the three stages in both the breeds (Supplementary Table S4). Most of the terms were enriched at the first stage, being 44 for BM1and 40 for TN1. In contrast, much less terms were enriched at the latter two stages: 2 for BM4, 7 for TN4, 8 for BM6, and 5 for TN8.

At the first stage, GO terms are mainly involved in metabolic process and development process for both the breeds. In addition, GO terms were also involved in cell proliferation, cell death, etc. for BM1, and reproductive process and sexual reproduction for TN1. At the second stage, BM4 was represented by only two GO terms of behaviour and lymphocyte co-stimulation. For TN4, however, most of the enriched terms were related to response to stimuli. At the third stage, GO terms represented at BM6 were mainly related to organism development, and terms related to organism movement were enriched for TN8 (Supplementary Table S4).

### 3.5. Expression differences between the two breeds

A total of 1600 BHU and 759 THU genes were identified, and then were enriched in 74 and 16 GO terms involving in biological process, respectively (Supplementary Table S5). The 74 GO terms for Bama pig mainly referred to the development process, such as multicellular organismal development (204), anatomical structure development (192), cellular developmental process (130), and anatomical structure morphogenesis (120), negative regulation of biological process (115), followed by regulation of biological process, such as positive regulation of biological process (149), regulation of biological quality (126), positive regulation of cellular process (133), and response to stress (109). However, The 16 GO terms for Tibetan pig mainly involved in stimuli response, such as response to stress (45), system process (35), and response to external stimulus (25).

### 3.6. Hormone genes expression pattern in postnatal pig pituitary

**Figure 3.**
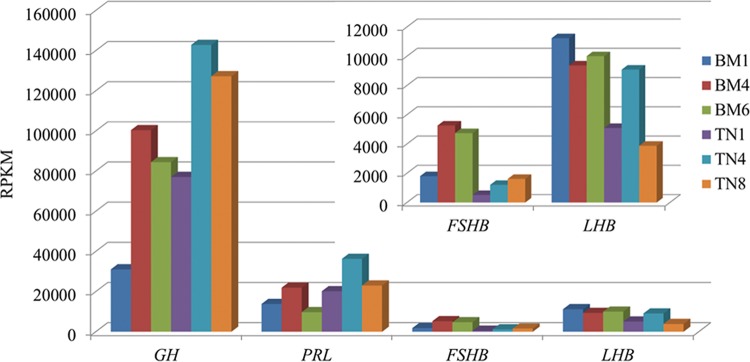
The expression levels of four anterior pituitary hormones in the six libraries measured in RPKM.

The four most highly expressed hormone genes (*GH*, *PRL*, *LHB*, and *FSHB*) exhibited three different expression patterns among the three stages in the two breeds (Fig. 3). Both *GH* and *PRL* genes were expressed as a low–high–low pattern. The expression level of *GH* in Bama pig increased with ca 3.23-fold, up to 100384.4 RPKM at BM4, and then decreased to ca 0.84-fold at BM6, and similarly in Tibetan pig. Similar pattern of gene activity change was observed in the *PRL* gene as the *GH* gene between the three stages within breed. However, *FSHB* and *LHB* gene showed different expression patterns between the two breeds: for *FSHB* gene, it displayed a low–high–low pattern in Bama pig and a gradient of increasing expression pattern in Tibetan pig; for *LHB* gene, it exhibited a high–low–high expression pattern in Bama pig and a low–high–low expression pattern in Tibetan pig (Supplementary Table S6).

### 3.7. SNP identification

We identified 19 996, 15 964, and 2213 SNPs in the sequence comparisons of Bama pig versus Duroc, Tibetan pig versus Duroc, and Bama pig versus Tibetan pig, respectively (Table 3; Supplementary Table S7). The largest proportion (34.2%) of SNPs was found in intergenic regions. Around 19.5% of the SNPs were located in intronic regions, 26.4% in coding regions, 9% in the 3′-UTR and 1.8% in the 5′-UTR. In addition, ca 1.3% of the SNPs were found in the 500-bp upstream from the translation start site, which was much lower than that in the 500-bp downstream from the stop codon (7.8%). In the comparison of Bama pig versus Duroc, the total numbers of non-synonymous and synonymous SNPs were 2271 and 2732, respectively, with ratios of 1:1.2 and 1.47:1 for the Bama pig versus Duroc, Tibetan pig versus Duroc, respectively, while 4.4:1 for Bama pig versus Tibetan pig.

**Table 3.** Summary of SNPs distribution in the *S. scrofa* genome

Genome regions	BM versus DK	TN versus DK	BM versus TN
CDS	5003	3769	675
Synonymy SNPs	2732	1528	125
Non-synonymy SNPs	2271	2241	550
Intergenic	7346	5576	684
Intron	4012	3929	303
5′UTR	336	238	52
3′UTR	1658	1212	249
TSS Up	250	231	24
TTS Down	1391	1009	226
Total	19 996	15 964	2213

BM, Bama pig; TN, Tibetan pig; DK, Duroc; CDS, coding sequence; 5′UTR, five prime untranslated region; 3′UTR, three prime untranslated region; TSS Up, 500 bp upstream of the transcription start site; TTS Down, 500 bp downstream from the transcription terminal site.

## 4. Discussion

We present here, to the best of our knowledge, the first comprehensive exploration of gene expression dynamics in the pituitary gland of miniature pig as implemented by RNA-seq technology. We found the proportions of uniquely mapped reads located in introns (27.47–42.78%) and splice junctions (14.01–19.59%) was much higher than that in other species.^14,32^ Most of the DEGs were identified at the first stage than the latter two stages, and the gene functions were also represented at BM1 and TN1 by cellular process and reproductive process, respectively. The number of SNPs identified between the two miniature breeds and Duroc was much larger than that within the two miniature pigs.

In this study, we found that 24.84% of the total high-quality paired-end reads (ca 246 millions) were filtered as unmatched, single-end matched, and bad matched reads, which could be due to the low sequence coverage of the swine reference genome (0.66×),^33^ reference genome errors, sequencing errors, and the mapping-defined criteria. Compared with that in other mammalian,^13,14^ a much larger proportion of uniquely mapped reads falls into the annotated intronic (on average: 33.07%) and intergenic (15.71%) regions, which suggested that the swine reference genome are far from being fully annotated. However, our transcriptome data have covered a large portion of the annotated transcripts (76.8%, 15 715/20 460) at Ensembl database (http://hgdownload.soe.ucsc.edu/goldenPath/susScr2/chromosomes/), indicating the robust ability of RNA-seq in discovering transcripts with lowly expressed values.^34^ The proportion (90.33%) of transcripts common in the two breeds is larger than that common in the stages within each breed (BM, 82.84%; TN, 83.28%), implying that the transcriptome variability derived from the different developing stages is much higher than that from the breeds. This is in accordance with the results from previous studies,^35–37^ which showed that a much larger fraction of transcriptome variability is explained by the tissue difference rather than the breed.

GO analysis for the co-expressed genes showed that most of the genes are involved in metabolic process and cellular process of biological process, the cell part and organelle of a cellular component, and the binding and catalytic activity of molecular function. Similar results are found in the transcriptome analysis of European eel,^38^ rainbow trout,^39^ silver carp,^40^ and muscle and ovary of a landrace female pig.^20^ A greater number of DEGs were upregulated at the first stage than that at the latter two stages in both the breeds, showing the pituitary gland was undergoing vigorous development process at the first stage. This finding was confirmed by the results derived from GO analysis, which showed that the first stage was greatly over-represented by the GO terms related to metabolic process and development process. The expression of BHU and THU genes illustrated the differences between the two miniature breeds. Bama pig was dominated by the expression of genes referred to organism development, whereas Tibetan pig was dominated by the expression of genes involved in response to stress. We also observed a larger number of BHU genes than that of THU genes, indicating that more genes were highly expressed in Bama pig than that in Tibetan pig.

Four hormone genes (*GH*, *PRL*, *LHB*, and *FSHB*) recurrently appeared in all the six sequencing libraries with extremely high expression levels. Any abnormality in the production of these hormones will cause isolated anterior pituitary hormone deficiencies and combined pituitary hormone deficiencies,^41,42^ implying their pivot roles in forming the molecular basis of the tissue specificity of the pituitary gland and enabling the normal development of different parts of the body. The *GH* gene, which is in critical need of normal growth of an individual, showed a postnatal growth wave peak at the second stage (BM4 and TN4) in both the breeds. This is in accordance with earlier observations that the inflexion point of growth curve of body weight in Bama pig was during 15–18 weeks in age,^43^ and the fast growth rate in the muscle fiber area of Tibetan male pig was reported in the 4th month compared with other months (2nd, 3rd, 5th, and 6th month).^44^ The *PRL* gene, which was reported to have the ability to stimulate mammary growth, reproduction, and lactation,^45^ showed a similar expression pattern as the *GH* gene, further confirmed that the intensive growth performance happens in ca the 4th month after birth. The different expression patterns of *FSHB* and *LHB* gene between the two pig breeds may be dependent on their stages of pubertal development, suggesting an earlier age of pubertal maturation in Bama pig than that in Tibetan pig. However, this observation is worthy of a further investigation like the profiling of plasma LHB and FSHB levels in peri-pubertal stages of the pigs.

A larger numbers of SNPs were found between Bama pig (or Tibetan pig) versus Duroc than that between Bama pig versus Tibetan pig, indicating the divergence between the miniature pig and the large pig is greater than that between the two miniature pigs. Nevertheless, it should be noted that we could not clearly determine how many SNPs are doubtless due to RNA editing, sequence errors (in spite of sequence quality filtration), mapping error, or the reference sequencing errors.^32^

In conclusion, we have presented here so far the most complete transcriptome data available for pituitary gland along the postnatal developing stages in Chinese native miniature pig. We found a large fraction of transcriptome variability, which can be explained by the difference between postnatal developing stages rather than between breeds. We also noted that the differences might be the reflection of genomic variations of individual pigs and experimental variations (e.g. tissue extraction, RNA preparation, and library construction), although the possibilities should be minimal. The four most actively transcribed hormone genes are indicative of their important functions throughout the period of postnatal development. The differences between stages and between breeds can be illustrated by the stage- and breed-specific upregulated genes. The SNPs analysis revealed the greater divergence between the two miniature pig breeds and the large breed. These findings provide new insights into the postnatal developing process of the pig pituitary gland, and will facilitate further genomic and gene function researches on the miniature pig.

## Supplementary data

Supplementary data are available at www.dnaresearch.oxfordjournals.org.

## Funding

This study was supported by grants from the National Basic Research Program of China (2011CB944100) and the National Transgenic Breeding Project of China (2013ZX08009-003).

## Supplementary Material

Supplementary Data
