# Supplementary material for: Transcriptome Profiling Identifies Differentially Expressed Genes in Postnatal Developing Pituitary Gland of Miniature Pig
Source: DNA Res. 2013 Nov 26;21(2):207–16. doi: 10.1093/dnares/dst051 (PMC3989491; doi:10.1093/dnares/dst051)
Supplement: Supplementary Data [file supp_21_2_207__index.html]

Transcriptome Profiling Identifies Differentially Expressed Genes in Postnatal Developing Pituitary Gland of Miniature Pig — Transcriptome Profiling Identifies Differentially Expressed Genes in Postnatal Developing Pituitary Gland of Miniature Pig — Supplementary Data 

# Transcriptome Profiling Identifies Differentially Expressed Genes in Postnatal Developing Pituitary Gland of Miniature Pig

## Supplementary Data

Supplementary Data

**Files in this Data Supplement:**

- Supplementary Data - Doc file
- Supplementary Figure 1 - tif file
- Supplementary Figure 1Legend - doc file
- Supplementary Table 1 - xls file
- Supplementary Table 2and6 - doc file
- Supplementary Table 3 - xls file
- Supplementary Table 4 - xls file
- Supplementary Table 5 - xls file
- Supplementary Table 7 - xls file
