# Supplementary material for: Transcriptome Profiling Identifies Differentially Expressed Genes in Postnatal Developing Pituitary Gland of Miniature Pig
Source: DNA Res. 2013 Nov 26;21(2):207–16. doi: 10.1093/dnares/dst051 (PMC3989491; doi:10.1093/dnares/dst051)
Supplement: Supplementary Data [file supp_dst051_dst051supp.doc]

**Supplementary Data and Information**

**Information about the two miniature pig breeds**

The Bama pig was developed naturally in Chinese remote mountainous areas in Guangxi Province with altitudes of 600 - 800 meters above sea level (m. a. s. l.). The breed is genetically stable, highly inbred, inexpensive and small. These advantages make the breed to be useful for comparative medicine researches in China since the 1980s, especially as a prospective animal model in preclinical drug evaluation.[^3-6^](#_ENREF_3)

The Tibetan pig is another Chinese indigenous breed, which have originated and were mainly distributed in the Tibetan highlands,[^7^](#_ENREF_7) the largest continuous high-altitude ecosystem in the world with an average altitude of more than 4000 m.a.s.l.. This breed is characterized for its small body size, very good vision, sensitive smell, highly developed respiratory system, well-developed heart, high deposition of fat and low growth rate, which have allowed it to adapt to the extreme conditions like hypoxia in Tibet. For its remarkable advantages over lowland pigs, the breed has been increasingly used as an experimental model in medicine research as well. In addition, many experimental animal centers and institutes have used them as a standardized experimental animal.[^8-10^](#_ENREF_8)

**Supplementary Figure Legends**

**Supplementary Figure S1.** Number of up-regulated DEGs [at least 2 mapped reads, *P*-value ≤ 0.001 and the absolute values of log_2_(Ratio ≥ 1)] in the three developmental stages. Infancy: BM1 *vs*. TN1; puberty: BM4 *vs*. TN4; adulthood: BM6 *vs*. TN8.

**Supplementary Tables**

**Supplementary Table S1**. Gene annotation in all of the six samples with RPKM values.

**Supplementary Table S2.** Summary of the number of genes detected in the two breeds across three stages (RPKM>0).

**Supplementary Table S3**. Sixty-one most highly transcribed genes and their expression values (RPKM) in the six samples.

**Supplementary Table S4**. Gene ontology (GO) analysis with the stage-specific highly up-regulated. DEGs involved in biological process at each stage with *P*-value ˂0.05.

**Supplementary Table S5.** Gene ontology (GO) analysis with the BM/TN-specific highly up-regulated (BHU/THU) genes involved in biological process with *P*-value ˂0.05.

**Supplementary Table S6.** The expression levels and fold change ratio of the hormone gene among stages and between breeds based on RPKM values.

**Supplementary Table S7.** Distribution of SNPs in the *S.scrofa* genome.

**Supplementary References**

1. Wu, F. C., Wei, H. and Wang, A. D. 2000, Analysis of Genetic Constrution of Population in Bama Miniature Pigs by RAPD. *Acta Lab. Sci. Sinica*, **8**, 235-240.

2. Shang, H. T., Niu, R., Wei, H., et al. 2001, Genetic analysis of 35 microsatellite loci in three miniature pig breeds. *Yi Chuan*, **23**, 17-20.

3. Liu, Y., Zeng, B. H., Shang, H. T., Cen, Y. Y. and Wei, H. 2008, Bama Miniature Pigs (Sus scrofa domestica) as a Model for Drug Evaluation for Humans: Comparison of In Vitro Metabolism and In Vivo Pharmacokinetics of Lovastatin. *Comp. Med.*, **58**, 580-587.

4. Li, J., Liu, Y., Zhang, J. W., Wei, H. and Yang, L. 2006, Characterization of hepatic drug-metabolizing activities of Bama miniature pigs (Sus scrofa domestica): comparison with human enzyme analogs. *Comp. Med.*, **56**, 286-290.

5. Liu, Y., Chen, J. Y., Shang, H. T., et al. 2010, Light microscopic, electron microscopic, and immunohistochemical comparison of Bama minipig (Sus scrofa domestica) and human skin. *Comp. Med.*, **60**, 142-148.

6. Shang, H., Yang, J., Liu, Y. and Wei, H. 2009, Tissue distribution of CYP3A29 mRNA expression in Bama miniature pig by quantitative reverse transcriptase-polymerase chain reaction (RT-PCR). *Xenobiotica*, **39**, 423-429.

7. Yang, S., Zhang, H., Mao, H., et al. 2011, The local origin of the Tibetan pig and additional insights into the origin of Asian pigs. *PLoS One*, **6**, e28215.

8. Li, J. Y. and Luo, Z. 1993, Research on the habits and characteristics of Tibet pigs on Tibet plateau. *Ecol. Domestic Anim.*, **14**, 18-22.

9. Li, H. T., Zhang, X. F., Wu, Q. H., Liu, Y. Z., Ren, L. H. and Gu, W. W. 2008, Comparative study on growth and development and reproduction traits of Tibet hogs in subtropics. *Heilongjiang Anim. Sci. Vet. Med.*, 14-16.

10. Esteban, M. A., Xu, J., Yang, J., et al. 2009, Generation of induced pluripotent stem cell lines from Tibetan miniature pig. *J. Biol. Chem.*, **284**, 17634-17640.
