# Supplementary figures and images for: Transcriptome Profiling Identifies Differentially Expressed Genes in Postnatal Developing Pituitary Gland of Miniature Pig
Source: DNA Res. 2013 Nov 26;21(2):207–16. doi: 10.1093/dnares/dst051 (PMC3989491; doi:10.1093/dnares/dst051)

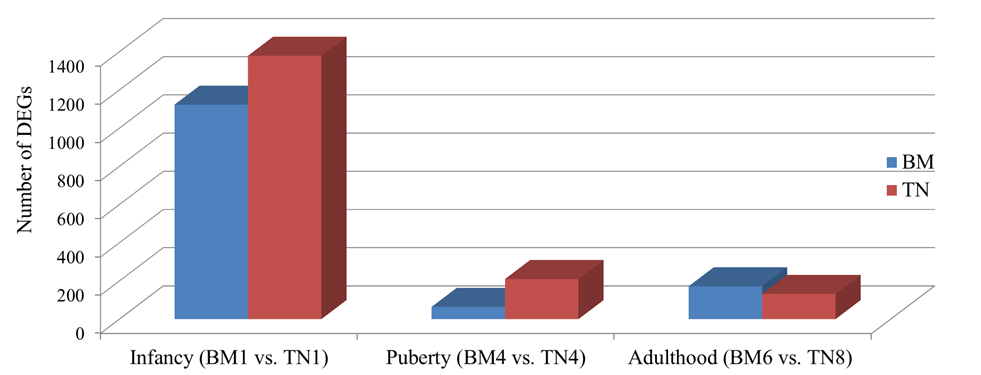

Supplement: Supplementary Data [file supp_dst051_dst051supp_fig1.tif]
