# Supplementary material for: Transcriptome Profiling Identifies Differentially Expressed Genes in Postnatal Developing Pituitary Gland of Miniature Pig
Source: DNA Res. 2013 Nov 26;21(2):207–16. doi: 10.1093/dnares/dst051 (PMC3989491; doi:10.1093/dnares/dst051)
Supplement: Supplementary Data [file supp_dst051_dst051supp_fig1Legend.doc]

**Supplementary Figure legends**

**Supplementary Figure S1.** Number of up-regulated DEGs [at least 2 mapped reads, *P*-value ≤ 0.001 and the absolute values of log_2_(Ratio ≥ 1)] in the three developmental stages. Infancy: BM1 *vs*. TN1; puberty: BM4 *vs*. TN4; adulthood: BM6 *vs*. TN8.
