# Supplementary material for: Transcriptome Profiling Identifies Differentially Expressed Genes in Postnatal Developing Pituitary Gland of Miniature Pig
Source: DNA Res. 2013 Nov 26;21(2):207–16. doi: 10.1093/dnares/dst051 (PMC3989491; doi:10.1093/dnares/dst051)
Supplement: Supplementary Data [file supp_dst051_dst051supp_table2and6.doc]

**Supplementary Table S2.** Summary of the number of genes detected in the two breeds across three stages (RPKM>0)

| **Classes** | **Number of genes** |
| --- | --- |
| Genes detected in the six samples | 15715 |
| Genes detected commonly in BM and TN | 14195 |
| Genes detected in all the six samples | 11844 |
| Genes detected in BM | 14780 |
| Genes detected in TN | 15130 |
| Genes detected in all three stages in BM | 12244 |
| Genes detected in all three stages in TN | 12601 |
| Genes detected in BM1 | 13249 |
| Genes detected in BM4 | 13286 |
| Genes detected in BM6 | 13887 |
| Genes detected in TN1 | 14132 |
| Genes detected in TN4 | 13623 |
| Genes detected in TN8 | 13739 |
| Genes detected specifically in BM1 | 421 |
| Genes detected specifically in BM4 | 312 |
| Genes detected specifically in BM6 | 649 |
| Genes detected specifically in TN1 | 604 |
| Genes detected specifically in TN4 | 356 |
| Genes detected specifically in TN8 | 407 |
| Genes detected commonly in BM1 and BM4 | 160 |
| Genes detected commonly in BM4 and BM6 | 570 |
| Genes detected commonly in BM1 and BM6 | 424 |
| Genes detected commonly in TN1 and TN4 | 431 |
| Genes detected commonly in TN4 and TN8 | 235 |
| Genes detected commonly in TN1 and TN8 | 496 |
| Genes detected exclusively in BM | 585 |
| Genes detected exclusively in TN | 935 |

**Supplementary Table S6.** The expression levels and fold change ratio of the hormone gene among stages and between breeds based on RPKM values

| **GeneName** | **Expression level in RPKM** | | | | | |  | **Fold-change ration** | | | |
| --- | --- | --- | --- | --- | --- | --- | --- | --- | --- | --- | --- |
|  | **BM1** | **BM4** | **BM6** | **TN1** | **TN4** | **TN8** |  | **BM4/BM1** | **BM6/BM4** | **TN4/TN1** | **TN8/TN4** |
| *GH* | 31069.72 | 77102.7 | 100384.4 | 142842.36 | 84450.43 | 127240.54 |  | 3.23 | 0.84 | 1.85 | 0.89 |
| *PRL* | 13842.91 | 20192.36 | 21952.78 | 36311.17 | 9751.84 | 23028.83 |  | 1.59 | 0.44 | 1.8 | 0.63 |
| *FSHB* | 1797.51 | 514.15 | 5261.15 | 1203.45 | 4741.96 | 1611.66 |  | 2.93 | 0.9 | 2.34 | 1.34 |
| *LHB* | 11228.79 | 5091.51 | 9371.22 | 9083.93 | 10001.14 | 3876.18 |  | 0.83 | 1.07 | 1.78 | 0.43 |
